# New Year - New Challenges - New Achievements

**DOI:** 10.5935/1678-9741.20160094

**Published:** 2016

**Authors:** Domingo M. Braile

**Affiliations:** 1 Editor-in-Chief - BJCVS

At the beginning of November, the Manager of the Sociedade Brasileira de Cirurgia Cardiovascular (SBCCV), Dr. Meryt Zanini, the Editorial Assistant Camila Sáfadi and I participated in the IX WORKSHOP OF SCIENTIFIC EDITING, a specific event for Scientific Editors and professionals working with Scientific Journals in our country.

Among the topics discussed, the concern with the financial maintenance of scientific journals was at the top of the discussions, considering that the budget crisis faced by the development agencies was a multiplier of this situation in constant aggravation.

Another point widely debated is what concerns the relevance of the work of the Reviewers, and eventual forms of recognition of this important work, in the Universities, Institutions, and especially regarding CAPES (Coordination for the Improvement of Higher Education Personnel).

I have been insisting and claiming for a long time that CAPES should recognize the voluntary work of the Editors, Reviewers and those who give their technical opinion on the articles, punctuating its performance, by the number of reviews and opinions issued, similar to the conduct adopted in relation to the published articles.

The counterpoint lies in the demands, which are increasingly greater in relation to the quality of the opinions, something that must be structured and justified, since they are absolutely essential for the improvement of the manuscript to be published.

This is something that should be done together with the author, aiming at improving the articles and, consequently, improving the scientific publications, opening the necessary quotations indispensable to increase the Impact Factor, which is our greatest goal.

During the 5 days of the event, we were pleased to note the great evolution presented by the Brazilian Journal of Cardiovascular Surgery (BJCVS), showing its improvement and, above all, its maintenance at the top of the scientific-editorial innovations.

Once again we have made sure that the whole process of changes that we have been going through in recent months was the right decision, because we were able to fulfill all the necessary requirements to become one of the leading publications in the surgical field.

We are part of the small number of scientific journals indexed in all the important International Databases. This is one of the reasons why we adopted a few years ago, the trend currently confirmed of the publication being done exclusively in English, in digital texts with all the facilities offered by this technology, allowing online consultations, hypertexts and the diffusion of our works around the globe where English is considered the universal scientific language.

We made efforts to join the ScholarOne editorial submission and management system, recognized and used by major journals, a measure that is already showing results by the number of manuscripts coming from overseas, which are some of the traditional nations in the areas of research and development.

We also have spared no effort to rapidly join social media, occupying a vanguard position still incipient in other Brazilian journals! We have witnessed a steady rise in BJCVS followers in our Facebook page and our Blog, and we are working to open up our range of influence when it comes to spreading ideas in this hotly contested area.

It is clear that in the near future, articles and scientific journals will use this tool as an immediate forum for discussions of the most varied and disruptive subjects of the specialty. Obstacles will always be a permanent challenge in our scientific path, however, we are optimistic that the BJCVS, with the collaboration of researchers, authors and those who voluntarily collaborate with the Editorial Board, will reach its goals soon, putting the Journal back at the top of scientific publications of the specialty. Commitment is the watchword. And, fortunately, it is a verb conjugated by all who, tirelessly, help the BJCVS.

## LET'S KEEP UP THE FIGHT!

With the issue 31.6, we are completing the second year in which the Brazilian Journal of Cardiovascular Surgery became a bimonthly publication, (an issue every two months) besides a supplement issue.

Difficulties and victories are part of our routine, but with each achievement the emotion and the feeling we have when we accomplish our goals encourages us to continue.

This is the right time to thank the Board and the SBCCV Members for their continued support and prestige.

We should also thank the Authors, Associate Editors and Reviewers of the BJCVS who keep our journal alive.

With great affection, I thank the dedicated collaborators of the SBCCV and the BJCVS, who struggle to ensure that everything is done in the most appropriate way, always willing to help with joy and enthusiasm.

The BJCVS wishes a merry Christmas with illuminated charity and compassion, renewing in each one of us the deep feelings of faith and hope.

May the New Year be our companion in joy and friendship that surrounds us in a great family, united in the ideal of improving humanity.

My warmest regards!

Domingo M. Braile ^1^Editor-in-Chief - BJCVS

